# Different Effects of Cisplatin and Transplatin on the Higher-Order Structure of DNA and Gene Expression

**DOI:** 10.3390/ijms21010034

**Published:** 2019-12-19

**Authors:** Toshifumi Kishimoto, Yuko Yoshikawa, Kenichi Yoshikawa, Seiji Komeda

**Affiliations:** 1 Faculty of Life and Medical Sciences, Doshisha University, Kyotanabe, Kyoto 610-0394, Japan; kishimotodoushisha@gmail.com (T.K.); yoshi2989@gmail.com (Y.Y.); keyoshik@mail.doshisha.ac.jp (K.Y.); 2 Faculty of Pharmaceutical Sciences, Suzuka University of Medical Science, Suzuka, Mie 513-8670, Japan

**Keywords:** gene expression, higher-order structure of DNA, single-molecule DNA observation, anticancer agent, platinum-based drug, cisplatin

## Abstract

Despite the effectiveness of cisplatin as an anticancer agent, its trans-isomer, transplatin, is clinically ineffective. Although both isomers target nuclear DNA, there is a large difference in the magnitude of their biological effects. Here, we compared their effects on gene expression in an in vitro luciferase assay and quantified their effects on the higher-order structure of DNA using fluorescence microscopy (FM) and atomic force microscopy (AFM). The inhibitory effect of cisplatin on gene expression was about 7 times that of transplatin. Analysis of the fluctuation autocorrelation function of the intrachain Brownian motion of individual DNA molecules showed that cisplatin increases the spring and damping constants of DNA by one order of magnitude and these visco-elastic characteristics tend to increase gradually over several hours. Transplatin had a weaker effect, which tended to decrease with time. These results agree with a stronger inhibitory effect of cisplatin on gene expression. We discussed the characteristic effects of the two compounds on the higher-order DNA structure and gene expression in terms of the differences in their binding to DNA.

## 1. Introduction

**Figure 1:**
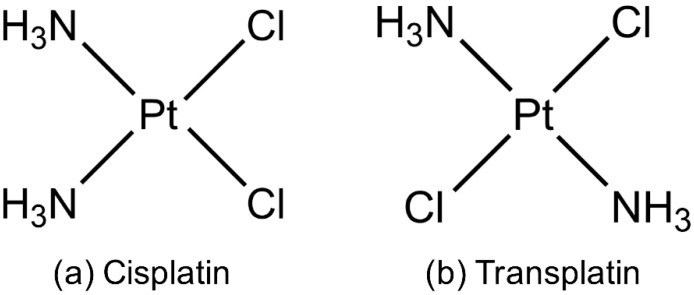
Chemical structures of the anticancer drug (**a**) cisplatin and its geometric isomer (**b**) transplatin.

Clinical use of cisplatin (*cis*-diamminedichloridoplatinum(II); see Figure 1) [1,2] started in the United States more than 40 years ago [3]. Cisplatin and related compounds, such as carboplatin (*cis*-diammine(1,1-cyclobutanedicarboxylato)platinum(II)) [4,5] and oxaliplatin (1*R*,2*R*-diaminocyclohexane)oxalatoplatinum(II)) [6,7], are called “platinum-based drugs” and are widely used as anticancer agents. DNA is believed to be the critical target of the platinum-based drugs [8,9]. The structure of these drugs can be described by a general formula, *cis*-[PtA_2_ X_2_], where A = ammonia or amine, and X = leaving group such as halide or carboxylate. In contrast to *cis*-Pt(II) complexes, the *trans*-Pt(II) derivatives have not entered a clinical trial yet. In particular, transplatin (*trans*-diamminedichloridoplatinum(II); see Figure 1), was found to be much less effective than cisplatin in blocking cell division in bacteria [10] and in inhibiting the growth of cancer cells [11]. Therefore, transplatin is known as a therapeutically inactive stereoisomer of cisplatin.

One possible explanation for the therapeutic inactivity of transplatin is that it undergoes more effective detoxification by sulfur-containing scavengers such as glutathione [12] and metallothionein [13]. Higher doses of transplatin as compared to cisplatin are required for binding of an equal number of Pt atoms per DNA nucleotide in living cells [14]. It was reported that the introduction of a sterically hindered planar amine ligand into transplatin instead of one of the ammonia ligands greatly enhanced cytotoxicity [15,16,17,18,19], probably because the bulky amine ligand decreased the rate of reaction of transplatin derivatives with glutathione. Another reason could be a different mode of interaction with DNA; the platinum(II) atom preferentially binds to guanine-N7, followed by adenine-N7, adenine-N1, and cytosine-N3 [20]. Although both cisplatin and transplatin form covalent DNA adducts, their structure is distinctively different. For instance, cisplatin forms mainly 1,2-intrastrand crosslinks (~80%) and rarely interstrand crosslinks (~1.5%) between two nucleobases in double-stranded DNA [21,22], whereas transplatin can hardly form intrastrand crosslinks but forms monofunctional adducts [23] and interstrand crosslinks [24] at a higher frequency than cisplatin. Local and second-order conformational changes induced in DNA by the two stereoisomers have also been discussed as possible reasons for their different antitumor efficacies [25,26,27,28]. 

Using fluorescence microscopy (FM) and atomic force microscopy (AFM), we elucidated the higher-order conformational changes of single long DNA strings induced by some novel cationic dinuclear Pt(II) complexes [29,30,31], which are potent anticancer drug candidates [32]. We found that these complexes shrink DNA much more efficiently than cisplatin. Here, we addressed the mechanism underlying the markedly different antitumor activity of cisplatin and transplatin by measuring changes in the higher-order structure of genome-sized DNA by FM and AFM. We found that the DNA-shrinking effect of cisplatin and transplatin correlated well with their potency as inhibitors of gene expression. We revealed that transplatin induced weak DNA shrinking just after its addition to DNA, and this effect almost disappeared after several hours, whereas the DNA-shrinking effect of cisplatin monotonously increased with time. Such marked difference in the effects of the two compounds could be interpreted in terms of the different chemical mechanisms of their interaction with DNA.

## 2. Results

### 2.1. Luciferase Assay for Gene Expression and Higher-Order DNA Structure

#### Inhibitory Effect on Gene Expression

**Figure 2:**
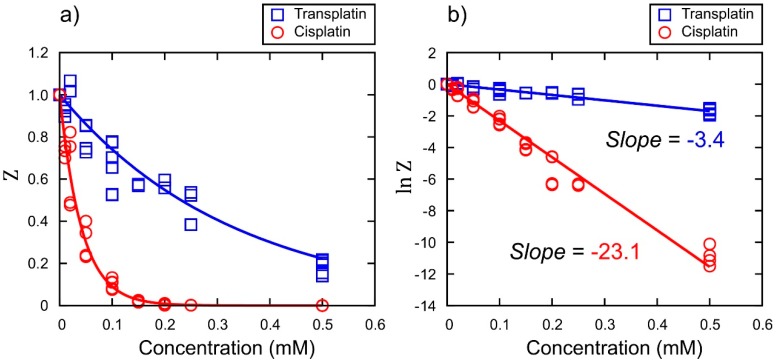
Effects of cisplatin and transplatin on gene expression efficiency. (**a**) The vertical axis is the relative emission intensity of the luciferin–luciferase reaction, Z, where the intensity in the absence of platinum complexes is taken as unity. (**b**) Quantification of the efficiency through the plots of lnZ vs. concentration using the same data as in (**a**).

To investigate the effects of cisplatin and transplatin on gene expression, we performed a cell-free luciferase assay. Figure 2a shows the relative luminescence intensity (Z) as a marker for gene expression at various concentrations of cisplatin or transplatin. The concentration dependence of the inhibition of gene expression over a certain time period τ (i.e., 90 min) was analyzed as follows. Assuming first order reaction kinetics for the decrease of the concentration of the active site of DNA, x, caused by the binding of a Pt chemical with the concentration c, we expect dx/dt ~−αcx, where α is the kinetic constant. Through integration, $x/x_{o}\sim \;e^{-\alpha ct\;}$ is deduced. Under the simple assumption, Z would be proportional to the total concentration of the product, luciferase. Thus, the dependence of Z on c for a reaction period of *τ* is given as $Z\;\sim \;e^{-\alpha c\tau \;}.$ From this relationship, the relative kinetic constant was evaluated from the slope of the graph: lnZ vs. c. A plot of the logarithm of the Z values vs. the concentrations of cisplatin or transplatin is shown in Figure 2b. The relative ratio of the slopes, −23.1 and −3.4, indicated that the inhibitory effect of cisplatin was about 7 times that of transplatin. 

### 2.2. Higher-Order Conformational Change in T4 Phage DNA

#### 2.2.1. FM Observations

**Figure 3:**
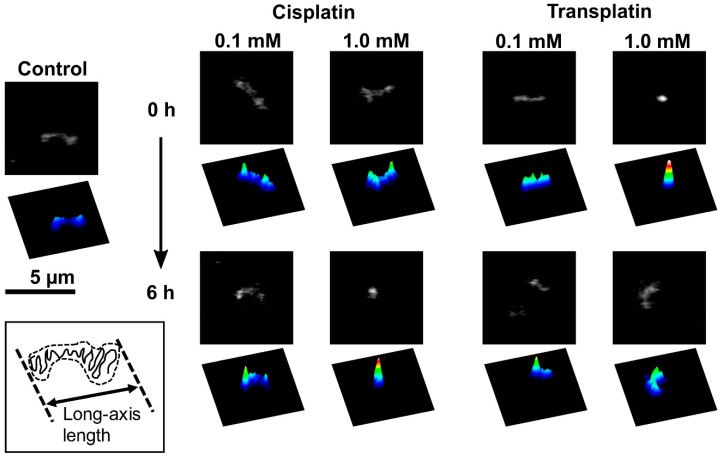
Fluorescence microscopy (FM) images of single T4 DNA molecules fluctuating in solution at 0.1 and 1.0 mM cisplatin or transplatin. Corresponding quasi-3D profiles are also shown. The DNA concentration was 1.5 µM in the nucleotide units.

To investigate the effects of cisplatin and transplatin on the higher-order structure of DNA, we used FM to observe single DNA molecules in the presence or absence of cisplatin or transplatin. Typical FM images and corresponding quasi-3D profiles of fluorescence intensity are shown in Figure 3. FM allows the observation of individual DNA molecules exhibiting translational and intramolecular Brownian motion in bulk solution. In the absence of cisplatin or transplatin, DNA molecules existed in an elongated coil state (Figure 3, control). Upon the addition of cisplatin, a partial globule conformation was generated (Figure 3, 0.1 mM cisplatin). An increase in cisplatin concentration or longer incubation time generated a compact globule conformation (Figure 3, 1.0 mM cisplatin, 6 h). In contrast, at 1.0 mM transplatin, the compact DNA was unfolded into a coil conformation with time.

**Figure 4:**
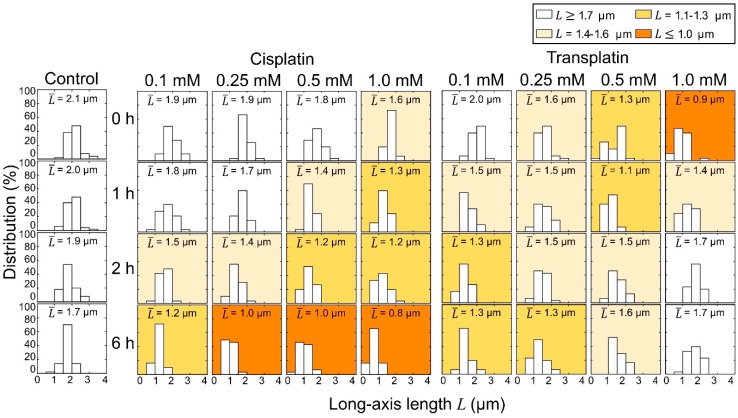
Distribution of the long-axis length L of T4 DNA observed by FM. At each condition, 50 DNA molecules were measured and the mean values, $\bar{L},$ are given in the boxes. The DNA concentration was 1.5 µM in nucleotide units.

**Figure 5:**
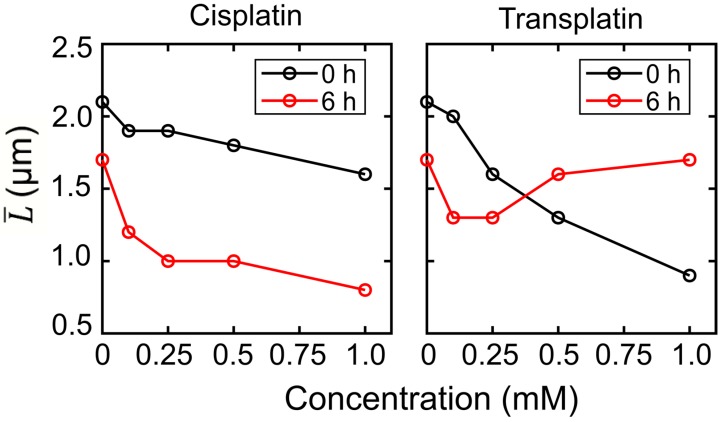
Average long-axis length $\bar{L}$ of T4 DNA vs. concentration of cisplatin or transplatin. The DNA concentration was 1.5 µM in the nucleotide units.

These observations clearly indicate that cisplatin and transplatin affect the higher-order structure of DNA by causing a shrunken state in a markedly different manner. Histograms of the long-axis length L of DNA as a function of the concentration of cisplatin and transplatin are shown in Figure 4. Both compounds shrank DNA molecules. In the presence of cisplatin, the degree of DNA shrinking increased with time. On the contrary, in the presence of transplatin, the DNA shrunken at 0 h gradually swelled, indicating a gradual decrease in the interaction between transplatin and DNA. To visualize the time-dependent effects of the two compounds, we plotted the average length $\bar{L}$ of DNA molecules at 0 h and 6 h against the compound concentration (Figure 5). Transplatin caused larger shrinking immediately after the addition compared to cisplatin. However, after several hours, DNA molecules swelled back in the presence of transplatin but not cisplatin. 

#### 2.2.2. AFM Observations

**Figure 6:**
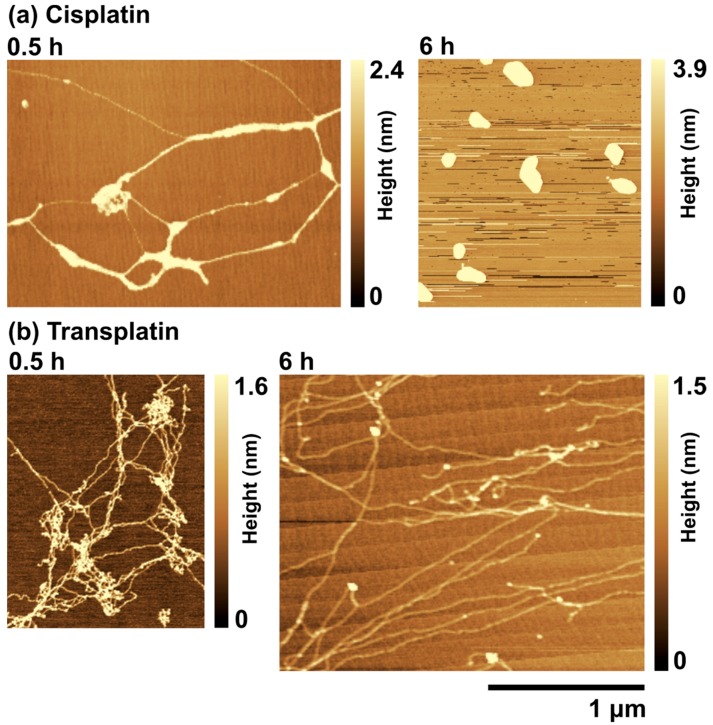
Atomic force microscopy (AFM) images of T4 DNA in the presence of (**a**) 0.5 mM cisplatin or (**b**) 0.5 mM transplatin. All specimens were gently adsorbed on the mica surface without shear stress and then dried. The DNA concentration was 1.5 µM in nucleotide units.

To investigate the effects of cisplatin and transplatin on the higher-order structure of DNA in more detail, we used AFM. Typical AFM images of T4 DNA in the presence of cisplatin and transplatin are shown in Figure 6. At 0.5 h, cisplatin induced inter- and intramolecular crosslinks in DNA, which resulted in bundles of parallel aligned DNA segments (Figure 6a). At 6 h, DNA became folded and its structure became condensed (Figure 6a). This change was consistent with the results of FM observations, suggesting that the bond between DNA and cisplatin becomes stronger with time. On the other hand, in the presence of transplatin, a mesh-like structure was observed at 0.5 h, which loosened with time (Figure 6b).

#### 2.2.3. Visco-Elasticity and Hydrodynamic Radius *R_H_* of the DNA Molecule

**Figure 7:**
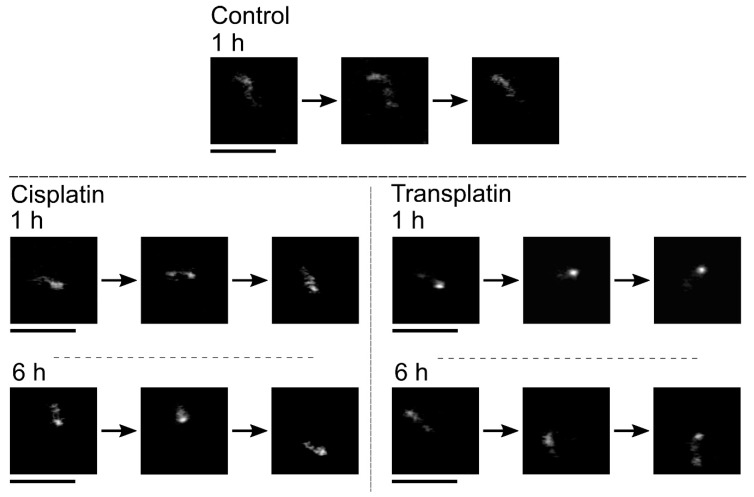
Intra-chain and translational Brownian motion of single T4 DNA molecules in bulk solution as observed by fluorescence microscopy. The time interval between neighboring frames is 0.1 s. Control: no cisplatin or transplatin. The concentration of cisplatin and transplatin was 0.5 mM. Scale bars, 5 µm.

**Figure 8:**
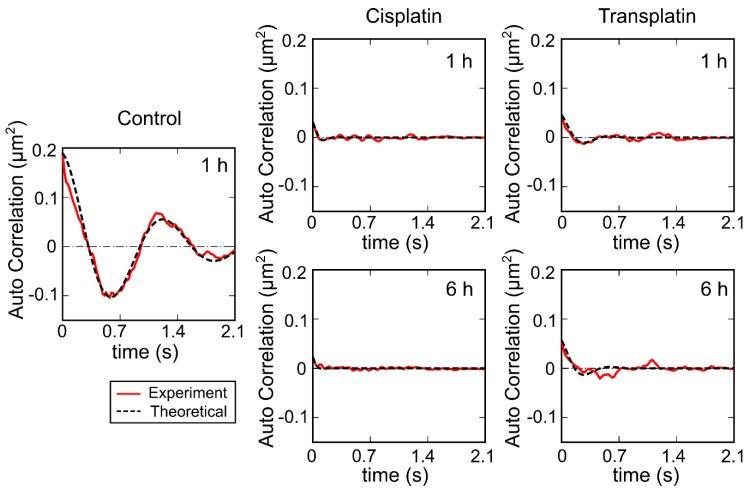
Autocorrelation function for the time-dependent fluctuation of the long-axis length of the single DNA molecules shown in Figure 7. Control: no Pt compounds. The concentration of cisplatin and transplatin was 0.5 mM. Solid lines, autocorrelation of DNA obtained from the time-dependent fluctuation of the long-axis length; broken lines, fitted curves based on Equation (2).

**Figure 9:**
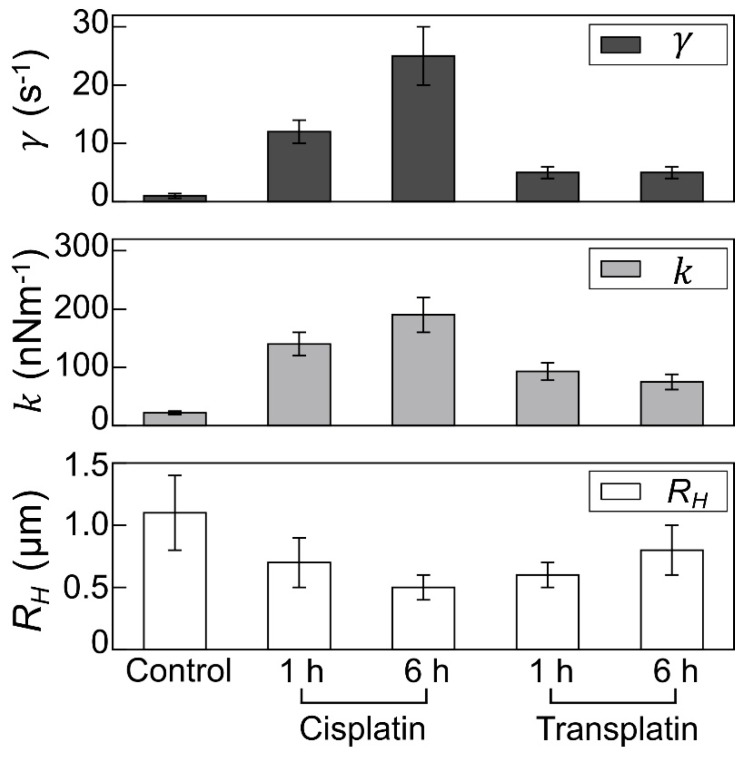
Visco-elastic properties and the hydrodynamic radius of DNA molecules. Upper and middle panels: Visco-elasticity of single T4 DNA molecules (166 kbp) in the presence of cisplatin or transplatin evaluated from the autocorrelation function of intrachain thermal fluctuation shown in Figure 8. k, spring constant; γ, damping constant. Lower panel: hydrodynamic radius *R_H_*. The *R_H_* values were calculated for at least 100 DNA molecules per sample. DNA concentration was 1.5 µM in nucleotide units. Concentration of cisplatin and transplatin was 0.5 mM.

To evaluate the intramolecular Brownian motion of DNA (Figure 7), we quantified the fluctuation of single DNA molecules in the fluorescence images [32,33,34,35]. Autocorrelation of DNA obtained from the time-dependent fluctuation of the long-axis length is shown in Figure 8, and the deduced spring and damping constants are summarized in Figure 9. In the control state, the spring constant was 2.2 ± 0.3 × 10^−8^ Nm^−1^ and the damping constant was 1 ± 0.4 s^−1^. By the addition of the platinum compounds, the spring constant increased greatly. The increase of spring constant for the cisplatin–DNA complex was more than 10 times that of the control DNA at 6 h, whereas the transplatin–DNA complex was 5 times that of the control DNA. Hence, the increase in the spring constant was found to be more significant in DNA with cisplatin than with transplatin. It was also noted that the spring constant for the transplatin-DNA complex tended to decrease from 1 h to 6 h, in contrast to the increasing tendency of the cisplatin-DNA complex. As revealed in Figure 9, the change in the viscosity constant indicated a similar trend to the change in the spring constant. In addition to the aforementioned analysis of the intrachain fluctuation (Figure 7, Figure 8 and Figure 9), we analyzed the translational motion to evaluate the *R_H_*. Although the fluorescence images do not give precise information on the actual sizes of individual DNA molecules because of the presence of a blurring effect of 0.3–0.5 μm [33,34,35], the analysis of the Brownian motion, or translational fluctuation, can provide the *R_H_* as a reliable measure of the size of individual DNA molecules. It was apparent that the effect on the hydrodynamic radius *R_H_* was observed in a very similar manner, although the hydrodynamic radius *R_H_* did not change greatly and the visco-elastic properties of the platinum compound–DNA complex changed more clearly. Cisplatin decreased the *R_H_* (Figure 9), and the effect became stronger with time, whereas the effect of transplatin was weaker at 6 h than at 1 h. 

## 3. Discussion

Inhibition of transcription is one of the mechanisms by which platinum-based drugs kill cancer cells [36]. The inhibition level appears to be related to the global DNA structure change induced by the formation of platinum–DNA adducts [37]. Therefore, the higher-order conformational change of DNA induced by each isomer might be related to its pharmaceutical activity or inactivity. There are no reports on the direct comparison of the higher-order structural changes induced in genome-sized DNA by cisplatin and transplatin. In this study, we used FM and AFM to monitor the structure of T4 phage DNA (166 kbp) at various concentrations of each platinum complex. When we previously monitored higher-order structural changes induced in DNA by cisplatin and a series of promising anticancer Pt(II) drug candidates [38], the inhibitory effects of these compounds on gene expression were correlated to the degree of DNA shrinkage: high DNA shrinkage resulted in a low expression level [19]. To shrink the DNA, it is necessary to neutralize the negative surface charge of the phosphate backbone, and the crosslinks between the segments along a DNA molecular chain, which also promotes DNA compaction [39,40]. In this study, both cisplatin and transplatin formed platinum(II)–DNA adducts, which neutralized the surface charge with positively charged Pt(II) ion and crosslinked DNA, and thus induced DNA shrinkage. The degree of shrinkage was higher in the presence of cisplatin than in the presence of transplatin after 6 h of exposure to the drug. The hydrolysis rate of cisplatin and transplatin affects their reactivity with biomolecules. Although trans effects between the two Cl ligands in transplatin appear to accelerate hydrolysis, by which a Cl ligand is displaced by a water molecule [41], it is difficult to determine the hydrolysis rate of which isomer is higher [42,43,44,45]. Indeed, reactions of each isomer with DNA are likely to provide comparable numbers of Pt atoms per DNA length in a cell-free medium [42,46,47]. Therefore, the different degrees of DNA shrinkage after 6 h are likely due to the different types of DNA crosslinks formed by cisplatin and transplatin, as discussed below. The inhibitory effect of cisplatin on gene expression was about 7 times that of transplatin. It has been reported that transplatin adducts are preferentially bypassed by RNA polymerase, and 4-fold more transplatin adducts than cisplatin adducts are required to similarly inhibit transcription [48]. It is also noted that similar effects of cisplatin on the shrinking of DNA has been reported for shorter DNA molecules [49,50].

Our most interesting finding came from FM and AFM observations over time. DNA molecules transiently became stiffer with a weak shrinkage immediately or 1 h after the addition of 0.5 or 1 mM transplatin and were softened back after several hours, whereas those in the presence of cisplatin continuously became stiffer and shrunken throughout the monitoring period. Compared to the control DNA molecules, a more significant shrinkage was observed in those with cisplatin than with transplatin. As mentioned in the Results (refer to Section 2.2.3), the spring and viscosity constants of DNA, which were evaluated from the time-dependent conformational fluctuation in solution observed through FM, reflect the effect of the platinum compounds on the physical property of their higher-order structure in a sensitive manner. As for the measurement of the elastic property of DNA, a number of publications have appeared, during the past couple of decades, that report the use of laser tweezers through the chemical modification with the binding of a micrometer-sized plastic sphere at the end of the DNA molecule (e.g., [51,52,53]). Unfortunately, most of these studies have reported the observed spring constant for DNA molecules shorter than several kbp, which is attributable to the technical difficulty of the measurement on individual giant DNA molecules. In the present study, we successfully applied our newly developed methodology for the analysis of intrachain Brownian motion that did not require the modification of DNA molecules longer than 100 kbp. It is expected that the application of the methodology on the measurement of the visco-elastic properties of giant DNA molecules adapted in the present study will open a new window for future studies on the relationship of the higher-order structure of genome-sized DNA with its biological functions.

**Figure 10:**
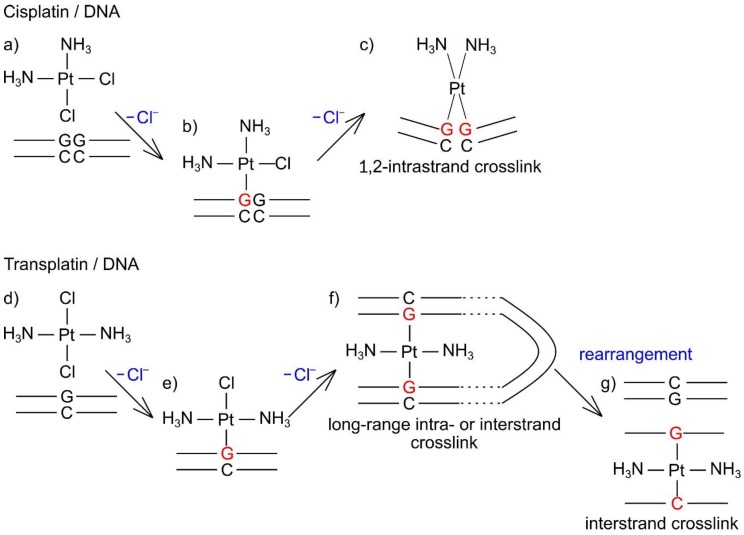
A supposed interaction mechanisms of cisplatin and transplatin with genome-sized DNA. (**a**) Unbound transplatin and DNA. (**b**) Cisplatin–DNA complex as a monofunctional adduct. (**c**) Formation of a cisplatin–DNA 1,2-intrastrand crosslink. (**d**) Unbound transplatin and DNA. (**e**) Transplatin–DNA complex as a monofunctional adduct. (**f**) Formation of a transplatin–DNA long-range intra- or interstrand crosslink. (**g**) Formation of a transplatin/DNA interstrand crosslink.

Using simulated and experimental data, Dutta et al. [54] showed the relation between the local and higher-order DNA structure upon the formation of cisplatin 1,2-intrastrand crosslinks. The different types of DNA adducts made by these isomers likely result in different higher-order structural changes of DNA. A supposed interaction mechanism of cisplatin and transplatin with genome-sized DNA is shown in Figure 10. Cisplatin forms bifunctional DNA adducts, such as 1,2-intrastrand (major type) and interstrand crosslinks [8,55,56,57,58,59], and induces severe distortion of the double helix in the local structure, resulting in shrinking and kinking of the higher-order DNA structure. On the other hand, because of steric restrictions, transplatin cannot form 1,2-intrastrand crosslinks and provides interstrand crosslinks at a higher frequency than cisplatin [24], and a relatively large proportion of transplatin–DNA adducts remain monofunctional [60]. The local conformational changes in DNA around a 1,2-intrastrand [55,58] or interstrand [59] crosslink made by cisplatin seem to be more severe than those around an interstrand crosslink made by transplatin [61,62] and its monofunctional DNA adducts [63]. Given that the degree of local DNA conformational changes can affect the higher-order structure, the transient shrinkage of DNA upon addition of transplatin seems unexpected. One possible explanation is the formation of long-range intra- or interstrand crosslinks between two nucleobases that are distant in the DNA primary structure but are spatially close to each other. 1,3-GNG intrastrand crosslinks (where G = guanine, N = adenine, cytosine, or thymine) can be formed in the reaction between transplatin and single-stranded oligonucleotides [60], and such DNA adducts can be readily rearranged into interstrand crosslinks after hybridization of platinated single strands with complementary strands [24,64,65,66]. Although 1,3-GNG intrastrand crosslinks are unlikely to form in double-stranded DNA [67], the long-range intra- or interstrand crosslinks looking like interhelix crosslinks could be formed between two proximal nucleobases when the negative DNA charge is partially neutralized by the formation of monofunctional adducts of transplatin. Accordingly, a partially folded DNA string might allow the monofunctional adducts to form bifunctional adducts of the long-range crosslinks, which can be rearranged to form energetically more favorable interstrand crosslinks between complementary GC base pairs [24]. This hypothesis is supported by the fact that transplatin is more reactive than cisplatin; that a relatively large proportion (~50%) of all adducts on the double-stranded DNA are monofunctional [23]; and that transplatin, owing to its geometry, is a useful tool to prepare a DNA triplex with interstrand GG or GC crosslinking [68,69].

## 4. Materials and Methods

### 4.1. Materials

Plasmid DNA (luciferase T7 control DNA, 4331 bp) containing a gene encoding luciferase and a T7 RNA polymerase promoter sequence was purchased from Promega (Madison, WI, USA). T4 GT7 phage DNA (166 kbp; contour length, 57 µm) was purchased from Nippon Gene (Toyama, Japan). The fluorescent cyanine dye YOYO-1 (1,10-(4,4,7,7-tetramethyl-4,7-diazaundecamethylene)-bis-4-(3-methyl-2,3-dihydro-(benzo-1,3-oxazole)-2-methylidene)-quinolinium tetraiodide) was purchased from Molecular Probes Inc. (Eugene, OR, USA). The antioxidant 2-mercaptoethanol was purchased from Wako Pure Chemical Industries (Osaka, Japan). Cisplatin was synthesized according to a previous report [70]. Transplatin was purchased from Sigma Aldrich (Saint Louis, MO, USA). Other chemicals were of analytical grade.

### 4.2. Methods

#### 4.2.1. Luciferase Assay for Gene Expression

The cell-free luciferase assay was carried out using a TnT T7 Quick Coupled Transcription/Translation System (Promega) similar to a previous report [71]. Plasmid DNA (4331 kbp) was used as a template. The final concentration of DNA was 1.5 μM in nucleotide units. The reaction mixture was incubated at 30 °C for 90 min in a Dry Thermo Unit (Taitec, Saitama, Japan). Luciferase expression was evaluated after the addition of luciferin (Luciferase Assay Reagent, Promega) by detecting the emission around 565 nm with a luminometer (Microtec Co., Chiba, Japan).

#### 4.2.2. FM Observations

T4 phage DNA was dissolved in 10 mM Tris-HCl buffer (pH 7.5) containing 4% (*v*/*v*) 2-mercaptoethanol and 80 mM KCl in the presence of various concentrations of cisplatin or transplatin. DNA concentration was 1.5 µM in nucleotide units. To visualize individual DNA molecules, 0.05 µM YOYO-1 was added to the DNA solution after incubation with the Pt complexes. DNA was imaged using an Axiovert 135 TV microscope (Carl Zeiss, Oberkochen, Germany) equipped with a 100× oil-immersion objective lens with fluorescent illumination from a mercury lamp (100 W) via a filter set (Zeiss-10, excitation BP 450–490; beam splitter FT 510; emission BP 515–565). A high-sensitivity EBCCD camera (Hamamatsu Photonics, Shizuoka, Japan) was used to observe individual DNA molecules. All images were recorded on a DVD at 30 frames per second and were analyzed with Cosmos image-analysis software (Library, Tokyo, Japan).

#### 4.2.3. AFM Measurements

For AFM imaging using an SPM-9700 microscope (Shimadzu, Kyoto, Japan), T4 GT7 phage DNA was dissolved (final concentration, 1.5 μM) in 10 mM Tris-HCl (pH 7.5) containing various concentrations of cisplatin or transplatin, incubated for 5 min, and then transferred onto a freshly cleaved mica surface (not pretreated with any cationic species) for 30 min at 25 °C. The mica was rinsed with pure water and dried under a gentle stream of nitrogen gas. All measurements were determined in air with tapping mode. A 200-μm long cantilever (OMCL-AC200TS-C2, Olympus, Tokyo, Japan) with a spring constant of 9 ± 20 N/m was used. The scanning rate was 0.4 Hz and images were captured on a 256 × 256 or 512 × 512 pixel format. The images were plane-fitted and flattened by the computer program equipped in the imaging module.

#### 4.2.4. Evaluation of Visco-Elasticity and Hydrodynamic Radius *R_H_* from the Analysis of DNA Molecule Fluctuations Observed by FM 

We evaluated the autocorrelation C(t) from the time-dependent fluctuation of the long-axis length, L(t) [33]:

(1) $$C\left(t\right)=\;\langle L\left(t\right)-\bar{L}\rangle \;\langle L\left(0\right)-\bar{L}\rangle$$

where $\bar{L}$ is the time-average of L.

Based on a theoretical model of thermal fluctuation for a microscopic visco-elastic object with a harmonic potential, the autocorrelation function is represented as in Equation (2) to a reasonable approximation:

(2) $$C\left(t\right)\;\sim \;\frac{k_{B}T}{k}e^{-\gamma t}\cos\omega t$$

(3) $$k\;\approx \;\frac{k_{B}T}{C\left(0\right)}$$

where $k_{B}$ is the Boltzmann constant, T is absolute temperature, k (N/m) is the spring constant, γ (1/s) is the damping constant, and ω (radian/s) is angular frequency. k is evaluated from the initial value of the autocorrelation function, C(0), by using Equation (3), where C(0) is the value of C(t) at t=0. Then, the value of γ is determined from the fitting curve.

The hydrodynamic radius of individual DNA molecules was evaluated from the time-dependent fluctuation of their center of mass in fluorescence images as described previously [32,34,35]. From the Brownian motion trails of the center of mass of DNA, we obtained the time dependence of the mean-square displacement of each molecule, and from these values we obtained the two-dimensional diffusion constant D as follows:

(4) $$\langle {\left(r\left(0\right)-r\left(t\right)\right)}^{2}\rangle =4Dt$$

where r is a two-dimensional positional vector of the center of mass of DNA molecules and the symbol t denotes a time average. In the actual analysis of the fluctuation, we used a modified equation [32,33,34,35] when a convectional flow was noticed. The hydrodynamic radius *R*_H_ was calculated from D based on the Stokes–Einstein relationship:

(5) $$R_{H}=\;k_{B}T/6\pi \eta D$$

where η is the viscosity of the solvent.

## 5. Conclusions

Using FM and AFM, we clearly delineated the difference between the effects of the geometric isomers cisplatin and transplatin on the higher-order structure of genome-sized DNA. The extent of DNA shrinkage was higher for cisplatin than for transplatin. This result agreed with a stronger inhibitory effect of cisplatin on gene expression in comparison with transplatin in a cell-free assay. Of note, transplatin induces DNA shrinkage transiently but more efficiently than cisplatin, probably because of the formation of long-range intra- or interstrand crosslinks between two nucleobases distant in the primary DNA structure but spatially close to each other, followed by a gradual rearrangement into an energetically preferable interstrand crosslink. This hypothesis was in line with a time-dependent rearrangement caused by transplatin on the order of several hours. Our finding of transient DNA shrinkage by transplatin was unprecedented and suggests that many undiscovered properties of genome-sized DNA molecules in the presence of biologically relevant compounds can be revealed by FM and AFM observations. We believe that our new experimental methodology to evaluate the visco-elastic properties of a single giant DNA molecule will be useful for future studies on the dynamic behavior of DNA molecules in solution. Additionally, AFM analysis of morphological changes of DNA at different time stages would be important and has been planned as our future project.

## Abbreviations

FM	Fluorescence microscopy
AFM	Atomic force microscopy
